# Safety and Efficacy of Direct Angioplasty in Acute Basilar Artery Occlusion Due to Atherosclerosis

**DOI:** 10.3389/fneur.2021.651653

**Published:** 2021-07-19

**Authors:** Gaoting Ma, Xuan Sun, Xu Tong, Baixue Jia, Xiaochuan Huo, Gang Luo, Bo Wang, Yiming Deng, Feng Gao, Ligang Song, Ning Ma, Zhongrong Miao, Dapeng Mo

**Affiliations:** Department of Interventional Neuroradiology, Beijing Tiantan Hospital, Capital Medical University, Beijing, China

**Keywords:** basilar artery occlusion, intracranial atherosclerotic disease, mechanical thrombectomy, stent-retriever thrombectomy, direct angioplasty

## Abstract

**Background and Purpose:** Endovascular treatment (EVT) is one of the promising treatment options in patients with intracranial atherosclerotic disease (ICAD)-related basilar artery occlusion (BAO). In this study, we compared the safety and efficacy of direct angioplasty (DA) with stent-retriever thrombectomy (SRT) with or without rescue treatment in ICAD-related BAO.

**Methods:** We retrospectively evaluated 187 patients who underwent EVT for BAO from January 2012 to July 2018. We identified patients who underwent EVT due to ICAD-related BAO. Patients who accepted SRT with or without rescue treatment were classified into the SRT group. Patients treated with DA with or without stent placement were classified into DA group. Clinical and laboratory findings and outcomes were compared between groups.

**Results:** A total of 108 patients were enrolled, among them 77 underwent SRT and 31 underwent DA; 61 (79.2%) SRT group patients underwent angioplasty with or without stent placement. Compared with patients in the SRT group, those in the DA group experienced a significantly shorter procedure time [60 min (60–120 min) vs. 120 min (60–120 min); *p* = 0.038] and a lower number of device passes [2 passes (1–2 passes) vs. 3 passes (2–4 passes); *p* < 0.001]. No significant differences in balloon angioplasty (35.5 vs. 22.1%; *p* = 0.150), emergent stent placement (64.5 vs. 57.1%; *p* = 0.481), successful recanalization (93.5 vs. 85.7%; *p* = 0.340), embolization in distal or new territory (3.2 vs. 9.1%, *p* = 0.314), and reocclusion (22.6 vs. 9.1%; *p* = 0.109) among DA and SRT groups were found. Additionally, no differences in symptomatic intracranial hemorrhage incidence [adjusted odds ratio (OR), 0.74; 95% CI, 0.06–9.44; *p* = 0.815], functional independence (adjusted OR, 1.44; 95% CI, 0.50–4.16; *p* = 0.497), and mortality rate (adjusted OR, 0.36; 95% CI, 0.06–2.04; *p* = 0.247) were noted among groups.

**Conclusions:** In certain patients with ICAD-related BAO, DA may shorten procedure time and reduce required device passes compared to SRT. In this study, DA was retrospectively found to be of similar safety and efficacy as SRT.

## Introduction

Acute ischemic stroke secondary to basilar artery occlusion (BAO) is associated with high rates of disability and mortality (1–3). The major pathomechanisms of BAO include *in situ* thrombosis over underlying intracranial atherosclerotic disease (ICAD) and embolization from distal sources (3). The proportion of ICAD-related BAO in prior studies was reported to be about 23–41% (3–6). Although the endovascular treatment vs. standard medical treatment for vertebrobasilar artery occlusion (BEST) study did not reveal any difference in favorable outcomes among patients who underwent endovascular therapy (EVT) compared to standard medical therapy alone, those findings may have been related to the early termination of the experiment due to cross-group recognition bias and poor patient compliance (7). Another recently published cohort study reported EVT to associate with a significantly better functional outcome than standard medical therapy among patients with acute BAO (8). Consequently, EVT remains among the most effective treatment options for such patients.

However, the best EVT strategy for patients with ICAD-related BAO remains controversial. The most frequently employed therapies include stent-retriever thrombectomy (SRT) and rescue treatment. The most commonly used rescue treatment is angioplasty (with or without stent placement); other rescue treatments include switching to another modality (e.g., from using a stent retriever to performing contact aspiration), intra-arterial thrombolysis (with alteplase or urokinase), and intravenous or administration of intra-arterial glycoprotein IIb/IIIa inhibitor (GPI) (9–11). However, several studies have reported rates of rescue treatment to be as high as 68.4–100% in the setting of ICAD-related BAO (6, 11, 12). Thus, in a selected patient population, superior treatment strategies are likely. The existing study highlights acute angioplasty as a technically feasible procedure. However, whether direct angioplasty (DA) for the treatment of ICAD-related BAO is as safe and efficacious as SRT remains unclear. In this study, we analyzed data from a clinical consecutive series of patients to assess both safety and efficacy profiles of DA in the treatment of acute ICAD-related BAO.

## Materials and Methods

### Patient Enrollment

**Figure 1:**
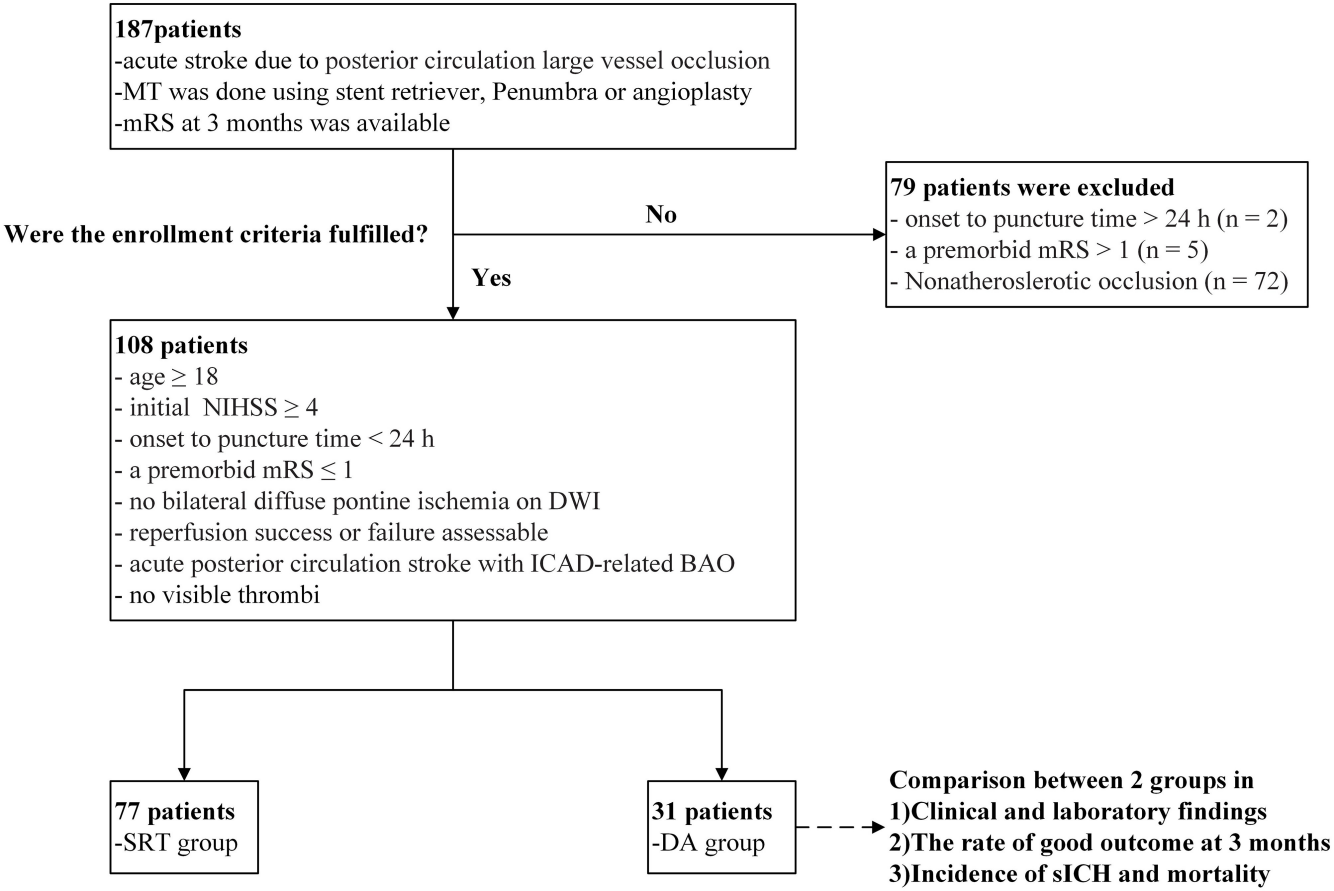
Flow chart of patient inclusion criteria and findings analyzed at each step.

We retrospectively evaluated the clinical data of patients who were diagnosed with acute stroke attributable to BAO and who consecutively underwent EVT at Beijing Tiantan Hospital from January 2012 to July 2018. Inclusion criteria were as follows: (1) age ≥18 years old; (2) baseline initial National Institutes of Health Stroke Scale score (NIHSS) ≥4; (3) onset-to-puncture time <24 h; (4) a premorbid modified Rankin Scale (mRS) score ≤ 1; (5) no bilateral diffuse pontine ischemia on diffusion-weighted imaging (DWI); (6) success or failure of assessable recanalization; (7) diagnosis of acute posterior circulation stroke with ICAD-related BAO; and (8) no visible thrombi. A flow chart of the patient selection process is shown in Figure 1. Cases in which the etiology of BAO was attributed to non-atherosclerotic conditions (e.g., cardiogenic embolism, artery-to-artery embolism, dissection, vasculitis, or moyamoya disease) were excluded from this study. The institutional review board approved this study and waived the requirement of informed consent for the inclusion of the study based on the retrospective nature of its design.

### Endovascular Treatment

**Figure 2:**
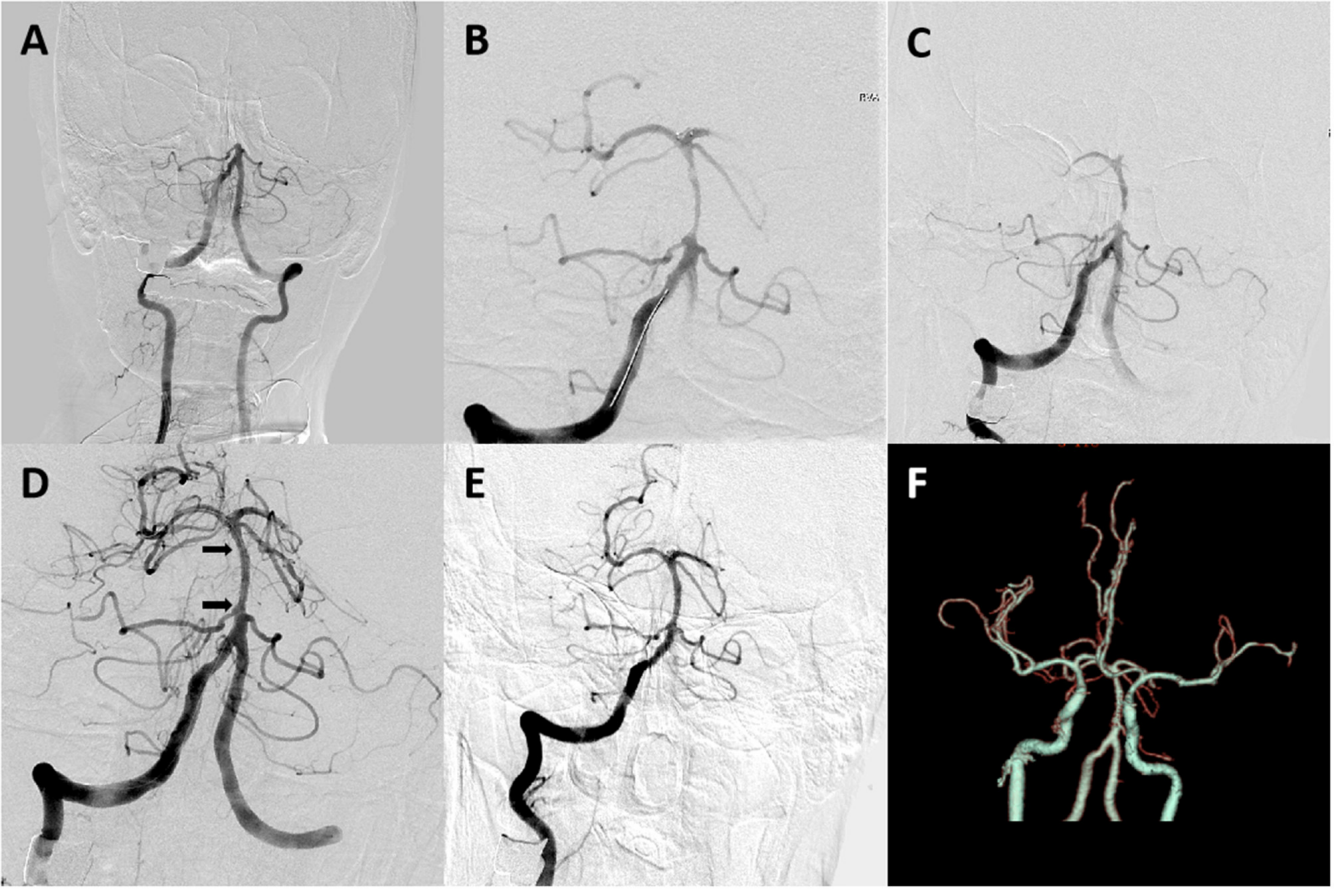
A 62-year-old male patient with coronary artery disease for 20 years and hypertension for 3 years presented with disturbance of consciousness for 4 h. **(A)** Digital subtraction angiography revealed occlusion at the proximal basilar artery segment. **(B,C)** Angiogram obtained after one Solitaire AB pass revealed severe underlying atherosclerotic stenosis of the proximal basilar artery segment. **(D,E)** Digital subtraction angiography after stent placement revealed good perfusion with fixed, focal basilar artery stenosis. Arrows indicate proximal and distal ends of the Apollo stent. **(F)** CT angiography on 24-h follow-up revealed a patent basilar artery. The patient had a modified Rankin scale (mRS) score of 3 on a 3-month follow-up.

**Figure 3:**
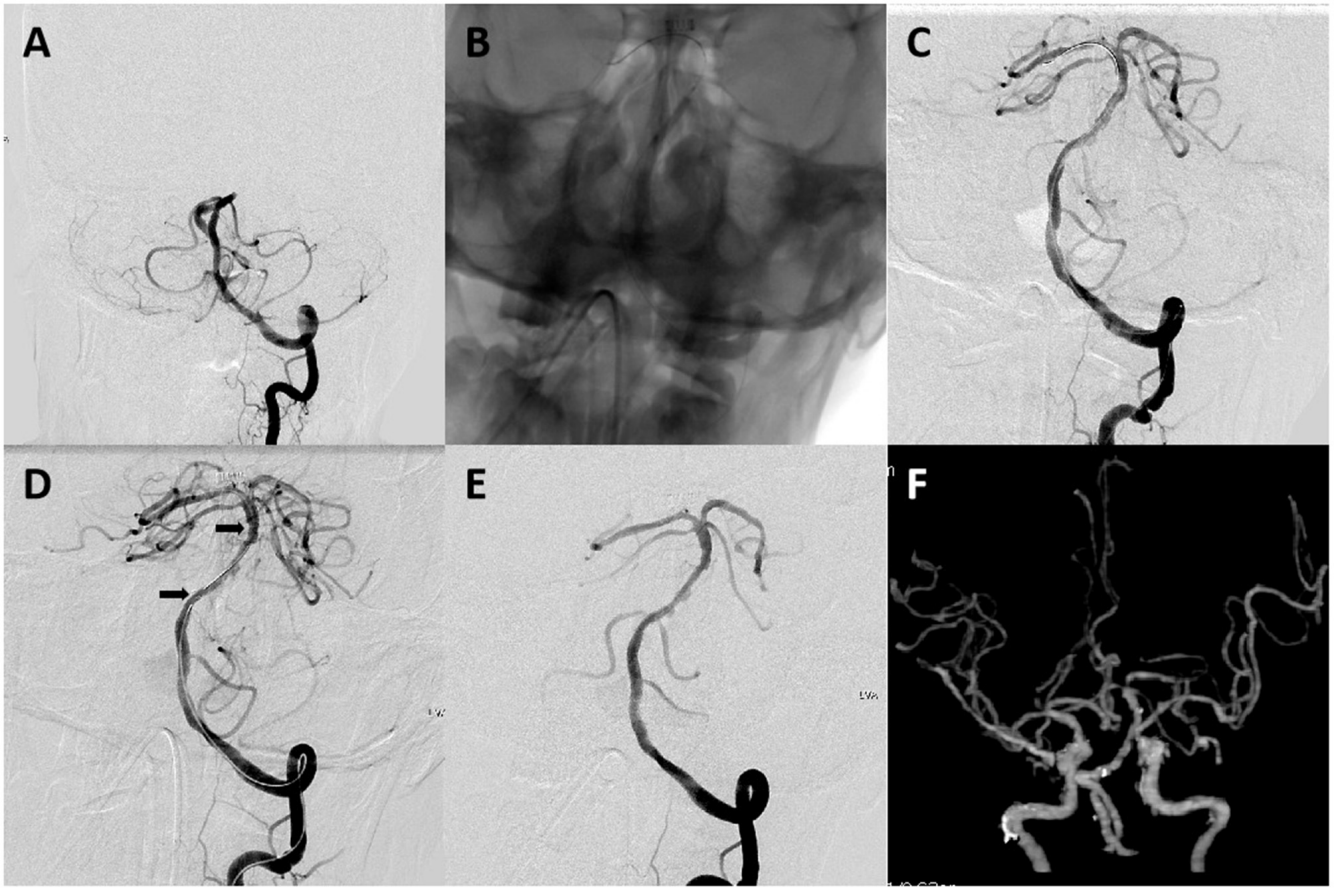
A 64-year-old male patient with acute stroke due to acute basilar artery occlusion. **(A)** Left vertebral artery angiogram revealed occlusion at the proximal basilar artery. **(B)** A 2 mm × 15 mm balloon (gateway) was deployed and inflated in the occluded segment. **(C–E)** Left vertebral artery angiogram obtained after intracranial angioplasty and stent placement revealed complete basilar artery recanalization and good distal perfusion. Arrows indicate proximal and distal ends of the Enterprise stent (4 mm × 22 mm). **(F)** CT angiography on 24-h follow-up revealed a patent basilar artery. The patient had a mRS score of 1 on a 3-month follow-up.

Endovascular treatment (EVT) was performed under local or general anesthesia in all patients. If the patient had a history remarkable for basilar artery stenosis or probable ICAD-related BAO was suggested on non-invasive angiography or catheter angiography, no visible thrombi were noted on angiography, and either SRT or DA was performed at the discretion of the treating neurointerventionalist. When underlying severe ICAD of the basilar artery was revealed on follow-up angiography after failed SRT, three different rescue treatment strategies were available, if necessary, namely, emergent angioplasty/stenting, intra-arterial thrombolysis (using alteplase or urokinase), or intra-arterial tirofiban infusion. Details of treatment techniques concerning both SRT and rescue therapy were described previously (11, 13). The DA technique, however, differed from standard endovascular methods. Initially, a microcatheter (0.018 or 0.021 in) was navigated over a microwire (0.014 in) to a position just beyond the occlusion site. Combined microcatheter and guiding catheter angiography was performed to document the length of the occluded segment. The microcatheter was exchanged over an extra-support exchange length microwire; a balloon was subsequently advanced into the occlusion site and inflated 1–3 times at 30 s each time. If successful recanalization was not achieved or if reocclusion manifested due to severe residual stenosis, rescue stenting was performed. Prior to stent deployment, a heparin bolus (3,000 IU) was administered intravenously. The type of stent (balloon- or self-expanding) was selected on the basis of both vascular characteristics and lesion morphology. Either low-dose intravenous or intra-arterial tirofiban (0.25–0.5 mg) was bolus-injected and maintained for 24 h, or an oral loading dose of aspirin and clopidogrel (300 mg each) was administered to patients prior to stent placement, according to the preference of neurointerventionist. All patients with stents were given clopidogrel (75 mg/day) and aspirin (100 mg/day) for 3 months. Figures 2, 3 detail how SRT and DA were performed.

### Outcome Measurement

The initiation of EVT was defined as the moment the needle punctured the common femoral artery. Procedure time was defined as the interval between the time of puncture and recanalization. Among both groups, all patients underwent non-enhanced CT or MRI scans immediately and 24 h after EVT. Follow-up vascular imaging was obtained in all patients at least once via MRI, CT, or digital subtraction angiography 1–90 days after treatment. Symptomatic intracerebral hemorrhage (sICH) was assessed by post-treatment CT or MRI scan and classified as either hemorrhagic infarction or parenchymal hemorrhage based on European Cooperative Acute Stroke Study (ECASS III) criteria (14). Successful recanalization was defined as achieving modified thrombolysis in cerebral ischemia (mTICI) grade 2b or 3 as confirmed on angiogram at least 10 min after recanalization. All images were analyzed retrospectively by two neurologists blinded to both patient data and study protocol.

Neurological evaluation was performed by a stroke neurologist immediately after treatment and 24 h and 3 months after treatment, when there were clinical changes and prior to the discharge of the patient. Functional outcome was assessed by a stroke neurologist using the mRS score via telephone interview 3 months after treatment. Functional independence was defined as a mRS score ≤ 2.

### Statistical Analysis

Baseline characteristics and treatment details were compared between DA and SRT groups. Successful recanalization, embolization in the new or distal territory, reocclusion, sICH, functional outcome at 3 months after treatment, and mortality were also compared between the groups. Multivariate analysis was performed with sICH, functional outcome, or mortality as dependent variables and with age, sex, baseline NIHSS, onset-to-puncture time, and tirofiban as covariates.

Medians (interquartile range: IQR) were used to summarize continuous data, and a two-sided *t-test* (for independent samples) or Mann-Whitney *U*-test was performed to detect differences between groups. Frequencies and percentages (%) were used to summarize binary data; between-group comparisons were performed using chi-squared or Fisher's exact tests, when appropriate.

All statistical analyses were performed with R (http://www.R-project.org, The R Foundation) and EmpowerStats (http://www.empowerstats.com, X&Y Solutions, Inc., Boston, MA). A *p* < 0.05 was considered significant.

## Results

Of the 108 patients, 77 (71.3%) patients underwent SRT therapy and 31 (28.7%) patients underwent DA therapy, respectively. In SRT group patients, stent-retriever devices used included the Solitaire AB/FR (74/77; 96.1%) and TREVO retriever (3/77, 3.9%). The Solitaire AB stent retriever was permanently detached in 16 patients, whereas Apollo and Wingspan stents were alternatively separately used in 24 and 16 patients, respectively. In the DA group, 11 (35.5%) patients underwent balloon angioplasty alone, whereas 20 (64.5%) patients underwent stent placement. Types of stents used in DA group patients were Wingspan (9), Apollo (7), and Enterprise (4).

**Figure 4:**
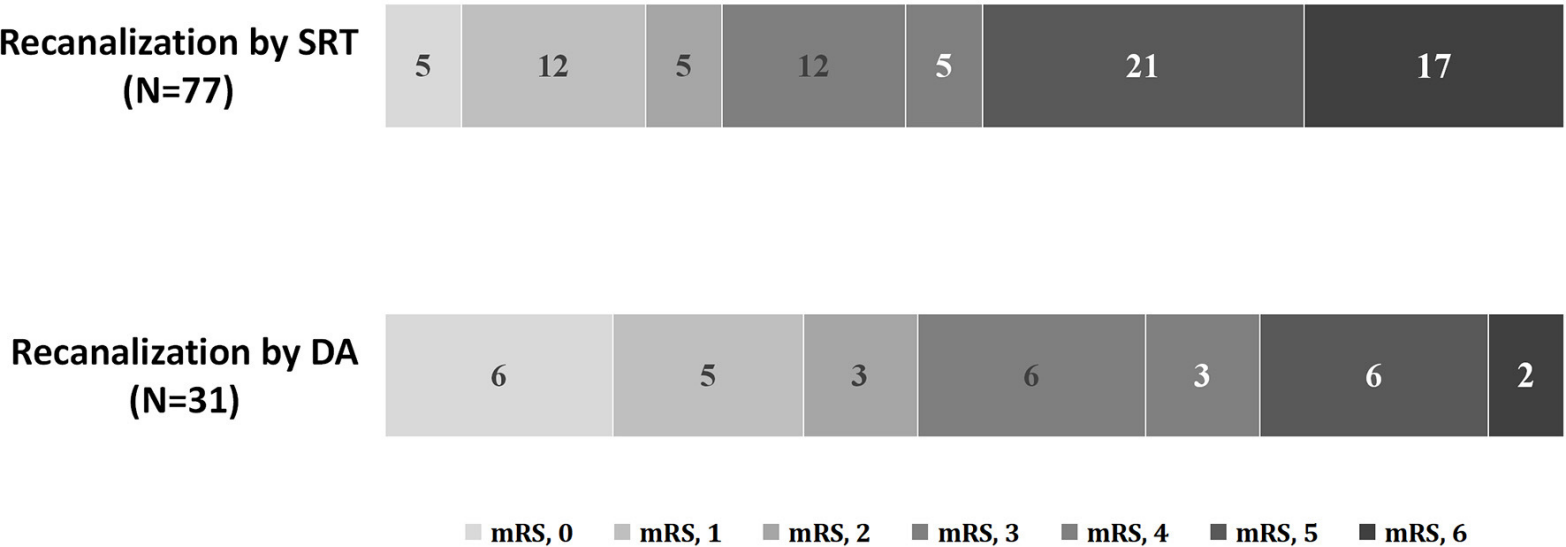
Distribution of 90-day mRS scores of patients with ICAD-related BAO. mRS, modified Rankin Scale; BAO, basilar artery occlusion; ICAD, intracranial atherosclerotic disease.

As shown in Table 1, no differences in clinical and laboratory findings except for age, baseline NIHSS score, and mechanical thrombectomy (MT) plus tirofiban use were found. Compared with patients in the SRT group, those in the DA group experienced a significantly shorter procedure time [60 min (60–120 min) vs. 120 min (60–120 min); *p* = 0.038] and a lower number of device passes [2 passes (1–2 passes) vs. 3 passes (2–4 passes); *p* < 0.001]. No significant differences in balloon angioplasty (35.5 vs. 22.1%; *p* = 0.150), emergent stent placement (64.5 vs. 57.1%; *p* = 0.481), successful recanalization (93.5 vs. 85.7%; *p* = 0.340), embolization in distal or new territory (3.2 vs. 9.1%, *p* = 0.314), and reocclusion (22.6 vs. 9.1%; *p* = 0.109) among DA and SRT groups were found. Additionally, no differences in symptomatic intracranial hemorrhage incidence [adjusted odds ratio (OR), 0.74; 95% CI, 0.06–9.44; *p* = 0.815], functional independence (adjusted OR, 1.44; 95% CI, 0.50–4.16; *p* = 0.497), and mortality rate (adjusted OR, 0.36; 95% CI, 0.06–2.04; *p* = 0.247) were noted among groups (Table 2). The 90-day comparison of mRS score shift among groups is detailed in Figure 4.

**Table 1:** Comparison of baseline data and treatment procedure outcomes among SRT and DA groups.

**Baseline characteristics, treatment procedures, and outcomes**	**All (*n* = 108)**	**SRT group (*n* = 77)**	**DA group (*n* = 31)**	***P*-Value**
Age, median (IQR), years	60 (53–64)	59 (52–63)	64 (55–71)	0.013
Male sex	94 (87.0)	67 (87.0)	27 (87.1)	>0.999
NIHSS score, median (IQR)	22 (12–34)	28 (13–35)	14 (7–29)	0.002
Pc-ASPECTS, median (IQR)	7 (5–8)	7 (5–8)	7 (6–8)	0.326
Pons-Midbrain Index, median (IQR)	2 (0–3)	2 (0–4)	2 (1–3)	0.475
Onset-to-puncture time, median (IQR), min	420 (300–600)	420 (300–570)	420 (300–600)	0.418
**Medical history**				
Hypertension	82 (75.9)	58 (75.3)	24 (77.4)	>0.999
Diabetes mellitus	27 (25.0)	18 (23.4)	9 (29.0)	0.625
Dyslipidemia	24 (22.2)	15 (19.5)	9 (29.0)	0.312
Current smoking	43 (39.8)	33 (42.9)	10 (32.3)	0.309
Coronary heart disease	8 (7.4)	4 (5.2)	4 (12.9)	0.223
Previous ischemic stroke	24 (22.2)	16 (20.8)	8 (25.8)	0.613
**Site of occlusion**				
Proximal BA	74 (68.5)	55 (71.4)	19 (61.3)	
Middle BA	33 (30.6)	21 (27.3)	12 (38.7)	0.541
Distal BA	1 (0.9)	1 (1.3)	0 (0.0)	
Good collateral (ASITN/SIR = 3–4)	11 (10.2)	7 (9.1)	4 (12.9)	0.726
Standard IV t-PA preoperative	21 (19.4)	15 (19.5)	6 (19.4)	> 0.999
General anesthesia	92 (85.2)	67 (87.0)	25 (80.6)	0.388
**MT procedure**				
Balloon angioplasty	28 (25.9)	17 (22.1)	11 (35.5)	0.150
Emergent stent placement	64 (59.3)	44 (57.1)	20 (64.5)	0.481
IA tPA or Urokinase	21 (19.4)	12 (15.6)	9 (29.0)	0.177
IV or IA tirofiban	87 (80.6)	67 (87.0)	20 (64.5)	0.014
**Treatment outcome**				
Procedural time, median (IQR), min	90 (60–120)	120 (60–120)	60 (60–120)	0.038
Number of device passes, median (IQR)	2 (2–3)	3 (2–4)	2 (1–2)	<0.001
Successful recanalization (mTICI 2b-3)	95 (88.0)	66 (85.7)	29 (93.5)	0.340
Distal or new territory embolization	8 (7.4)	7 (9.1)	1 (3.2)	0.314
Reocclusion	14 (13.0)	7 (9.1)	7 (22.6)	0.079

*Numbers are shown as median (IQR, interquartile range) or percentage (%). ASITN/SIR, American Society of Interventional and Therapeutic Neuroradiology/Society of Interventional Radiology; BA, basilar artery; DA, direct angioplasty; IA, intra-arterial; NIHSS, National Institute of Health Stroke Scale; IV, intravenous; mTICI, modified thrombolysis in cerebral ischemia; pc-ASPECTS, posterior circulation Alberta Stroke Program Early CT Score; SRT, stent-retriever thrombectomy; tPA, tissue-type plasminogen activator*.

**Table 2:** Multivariate regression analysis of effects of DA on sICH, functional outcomes and mortality.

**Outcome**	**SRT group**	**DA group**	***P-*value**	**OR (95% CI)**	**Adjusted *P-*value**	**Adjusted OR (95% CI)**
sICH^*^	4 (5.2)	1 (3.2)	0.663	0.61 (0.07–5.67)	0.815	0.74 (0.06–9.44)
3-mo mRS, 0–2^*^	22 (28.6)	14 (45.2)	0.101	2.06 (0.87–4.88)	0.497	1.44 (0.50–4.16)
3-mo mortality^*^	17 (22.1)	2 (6.5)	0.070	0.24 (0.05–1.13)	0.247	0.36 (0.06–2.04)

*CI, confidence interval; DA, direct angioplasty; mRS, modified Rankin Scale; OR, odds ratio; sICH, symptomatic intracerebral hemorrhage; SRT, stent-retriever thrombectomy*.

* *Adjusted for age, sex, baseline NIHSS, onset-to-puncture time, and tirofiban use*.

## Discussion

In this study, we found that patients who underwent DA experienced significantly shorter procedure time (60 vs. 120 min; *p* = 0.038) and fewer device passes (2 vs. 3; *p* < 0.001) than those who underwent SRT. No differences in sICH and favorable outcomes between the groups were found.

The estimated incidence of ICAD varied from 8.3 to 60%, depending on racial and geographical factors (15–17). Few studies have investigated the incidence of underlying ICAD in patients presenting with emergent large vessel occlusion (ELVO), especially with the posterior circulation that is affected. Compared to other stroke etiologies such as embolic occlusions, ICAD is less well-understood by physicians, and prior randomized trials have mostly studied anterior circulatory occlusion in Western populations. The proportion of ICAD-related BAO was reported to be 23–41% (3–6). Recent studies reported that ICAD was responsible for ~12–30.3% of patients with acute LVO who underwent endovascular thrombectomy in East Asia (9, 10). Severe ICAD is a major cause of treatment failure during thrombectomy in patients with acute LVO (18–20). Therefore, analyzing EVT strategies for ICAD-related BAO is highly warranted.

The treatment of patients with ICAD-related BAO with EVT typically involves two steps: initially, the performance of SRT to identify underlying culprit stenosis and, subsequently, administering rescue treatment to both remove the underlying stenosis and prevent reocclusion (20, 21). Rescue treatments (angioplasty with or without stenting) were reported to be essential for achieving successful recanalization in such patients in the setting of failed SRT (6, 9, 18, 20–22). In this study, 79.2% of SRT group patients (*n* = 61) underwent angioplasty with or without stenting as a rescue treatment. The main disadvantage of this strategy, however, is instant or early reocclusion of the target vessel due to severe stenosis (18, 23). Importantly, switching to another technique (i.e., from SRT to rescue treatments) after SRT failure leads to delays, and retrieval of stent retrievers distal to the stenosis frequently damages endothelium or the atherosclerotic plaque itself, thereby leading to an increased risk of acute thrombosis and reocclusion. This is likely because the use of a stent retriever predisposes to increased platelet activation and vessel dissection. These phenomena partly explain why the rate of GPI used among SRT patients was higher than that among DA patients (87.0 vs. 64.5%, *p* = 0.014). Complicated therapeutic strategies thus lead to longer procedure duration and poorer outcomes.

The advantages of emergent angioplasty are well-appreciated in the setting of acute myocardial ischemia. Approximately two decades ago, stenting began to replace angioplasty as the primary method of revascularization for the treatment of acute coronary syndrome (24–26). Although substantial differences in the typical etiology and vascular anatomy of stroke as compared to myocardial infarction exist, the pathogenesis of ICAD-related LVO is similar to that of myocardial infarction. Building on experience in treating and knowledge of the coronary intervention, the concept of angioplasty for acute stroke warrants serious consideration. One prospective study demonstrated that DA with stenting resulted in a 100% recanalization rate, although the study did not distinguish the mechanism of LVO (27). In a prospective registry [the Acute Basilar Artery Occlusion Study (BASILAR) study], a total of 10.2% (66/643) of subjects with BAO received DA as the first-choice treatment in the EVT group (8). In this study, we propose consideration of DA as a safe and technically feasible treatment strategy for a highly select group of patients with ICAD-related BAO.

In addition, a standardized periprocedural antiplatelet regimen for angioplasty with or without stenting due to ICAD in the setting of EVT of acute BAO was needed. Unfortunately, there are little data and a lack of consensus up to now. In a Delphi study, an oral loading dose of aspirin in combination with a P2Y12 inhibitor was suggested if a stent deployed during EVT (28). Recent studies indicated a more promising role of tirofiban as the periprocedural antiplatelet treatment of EVT (29, 30). Baek et al. reported that intravenous tirofiban was associated with a low reocclusion rate after emergent angioplasty with or without stenting in patients with ICAD-related large vessel occlusion stroke (31). In this study, the reocclusion rate in the SRT group was lower, which might be related to a higher percentage of patients receiving intravenous or intra-arterial tirofiban. Intriguingly, although the rate of reocclusion was higher in subjects treated with DA, the functional outcome was much better. Fewer pass numbers and shorter procedure time may be the underlying reason for this discrepancy.

Unless ICAD-related BAO can be diagnosed prior to MT, DA will remain as one of several available treatment options rather than a first-line strategy. Lin et al. found that opacification of the basilar tip, absence of convex edge, and long occlusion length on preprocedural CT angiography might be helpful in the diagnosis of ICAD-related BAO (32). Lee et al. reported the occluded vascular segment and the presence or absence of bilateral thalamic infarction to be helpful for predicting ICAD-related BAO (6). However, no effective method for identifying the etiology of BAO prior to MT has been proposed up to now. As such, future studies devoted to identifying ICAD-related BAO will likely provide greater treatment options in this subgroup of patients. Nevertheless, there is currently no valid reason to withhold DA from patients with a history remarkable for basilar artery stenosis and low clot burden.

## Limitations

This study had several limitations, including those inherent in a retrospective and uncontrolled study design. The selection of patients for stent placement depended on the preference of neurointerventionist. In particular, opinions concerning permanent stenting for failed SRT or DA treatment differed among the six involved physicians. In addition, this study was single centered and studied a relatively small sample size. Moreover, a special caution was needed when interpreting this result because all analyses were considered exploratory, especially the rate of functional outcome for 3 months in the DA group was much higher than that in the SRT group. A randomized, prospective trial is required to adequately assess the clinical efficacy of treatment modalities. Finally, angiograms were not reviewed by an independent core laboratory team blinded to clinical information.

## Conclusions

In certain patients with ICAD-related BAO, DA shortens procedure time and reduces device passes as compared to SRT. In this retrospective study, we found DA to be of similar safety and efficacy in a select group of patients as compared to SRT.

## Data Availability Statement

The raw data supporting the conclusions of this article will be made available by the authors, without undue reservation.

## Ethics Statement

The studies involving human participants were reviewed and approved by IRB of Beijing Tiantan Hospital, Capital Medical University. Written informed consent for participation was not required for this study in accordance with the national legislation and the institutional requirements. Written informed consent was obtained from the individual(s) for the publication of any potentially identifiable images or data included in this article.

## Author Contributions

DM and ZM designed, led the study and had full access to all of the data in the study, and took responsibility for the integrity of the data and the accuracy of the data analysis. GM prepared the first draft of the report. XT did statistical analyses. All authors except GM and BJ participated in patient enrolment and collection of data. All authors critically reviewed the report and approved the final version.

## Conflict of Interest

The authors declare that the research was conducted in the absence of any commercial or financial relationships that could be construed as a potential conflict of interest.

## Abbreviations

BAO: basilar artery occlusion
DA: direct angioplasty
DWI: diffusion-weighted imaging
EVT: endovascular treatment
ELVO: emergent large vessel occlusion
IART: intra-arterial recanalization therapy
ICAD: intracranial atherosclerotic disease
MI: myocardial ischemia
MT: mechanical thrombectomy
mTICI: modified Thrombolysis in Cerebral Ischemic
SR: stent retriever
SRT: stent-retriever thrombectomy.

## References

[1] Schonewille WJ Wijman CA Michel P Rueckert CM Weimar C Mattle HP. Treatment and outcomes of acute basilar artery occlusion in the Basilar Artery International Cooperation Study (BASICS): a prospective registry study. Lancet Neurol. (2009) 8:724–30. 10.1016/S1474-4422(09)70173-5 19577962

[2] Mortimer AM Bradley M Renowden SA. Endovascular therapy for acute basilar artery occlusion: a review of the literature. J Neurointerv Surg. (2012) 4:266–73. 10.1136/neurintsurg-2011-010090 21990530

[3] Mattle HP Arnold M Lindsberg PJ Schonewille WJ Schroth G. Basilar artery occlusion. Lancet Neurol. (2011) 10:1002–14. 10.1016/S1474-4422(11)70229-0 22014435

[4] Baek JM Yoon W Kim SK Jung MY Park MS Kim JT. Acute basilar artery occlusion: outcome of mechanical thrombectomy with Solitaire stent within 8 hours of stroke onset. AJNR Am J Neuroradiol. (2014) 35:989–93. 10.3174/ajnr.A3813 24335542 PMC7964531

[5] Gory B Eldesouky I Sivan-Hoffmann R Rabilloud M Ong E Riva R. Outcomes of stent retriever thrombectomy in basilar artery occlusion: an observational study and systematic review. J Neurol Neurosurg Psychiatry. (2016) 87:520–5. 10.1136/jnnp-2014-310250 25986363

[6] Lee YY Yoon W Kim SK Baek BH Kim GS Kim JT. Acute basilar artery occlusion: differences in characteristics and outcomes after endovascular therapy between patients with and without underlying severe atherosclerotic stenosis. AJNR Am J Neuroradiol. (2017) 38:1600–4. 10.3174/ajnr.A5233 28546252 PMC7960422

[7] Liu X Dai Q Ye R Zi W Liu Y Wang H. Endovascular treatment versus standard medical treatment for vertebrobasilar artery occlusion (BEST): an open-label, randomised controlled trial. Lancet Neurol. (2020) 19:115–22. 10.1016/S1474-4422(19)30395-3 31831388

[8] Writing Group for the BG Zi W Qiu Z Wu D Li F Liu H . Assessment of endovascular treatment for acute basilar artery occlusion via a nationwide prospective registry. JAMA Neurol. (2020) 77:561–73. 10.1001/jamaneurol.2020.0156 32080711 PMC7042866

[9] Kang DH Yoon W. Current opinion on endovascular therapy for emergent large vessel occlusion due to underlying intracranial atherosclerotic stenosis. Korean J Radiol. (2019) 20:739–48. 10.3348/kjr.2018.0809 30993925 PMC6470088

[10] Park H Baek JH Kim BM. Endovascular treatment of acute stroke due to intracranial atherosclerotic stenosis-related large vessel occlusion. Front Neurol. (2019) 10:308. 10.3389/fneur.2019.00308 31001193 PMC6454085

[11] Gao F Lo WT Sun X Mo DP Ma N Miao ZR. Combined use of mechanical thrombectomy with angioplasty and stenting for acute basilar occlusions with underlying severe intracranial vertebrobasilar stenosis: preliminary experience from a single Chinese center. AJNR Am J Neuroradiol. (2015) 36:1947–52. 10.3174/ajnr.A4364 26089317 PMC7965041

[12] Kim YW Hong JM Park DG Choi JW Kang DH Kim YS. Effect of intracranial atherosclerotic disease on endovascular treatment for patients with acute vertebrobasilar occlusion. AJNR Am J Neuroradiol. (2016) 37:2072–78. 10.3174/ajnr.A4844 27313131 PMC7963784

[13] Zhang X Luo G Mo D Ma N Gao F Zhang J. Predictors of good outcome after endovascular treatment for patients with vertebrobasilar artery occlusion due to intracranial atherosclerotic stenosis. Clin Neuroradiol. (2018) 29:693–700. 10.1007/s00062-018-0731-z 30498847

[14] Hacke W Kaste M Bluhmki E Brozman M Davalos A Guidetti D. Thrombolysis with alteplase 3 to 4.5 hours after acute ischemic stroke. N Engl J Med. (2008) 359:1317–29. 10.1056/NEJMoa0804656 18815396

[15] Bang OY. Intracranial atherosclerosis: current understanding and perspectives. J Stroke. (2014) 16:27–35. 10.5853/jos.2014.16.1.27 24741562 PMC3961814

[16] Holmstedt CA Turan TN Chimowitz MI. Atherosclerotic intracranial arterial stenosis: risk factors, diagnosis, and treatment. Lancet Neurol. (2013) 12:1106–14. 10.1016/S1474-4422(13)70195-9 24135208 PMC4005874

[17] Qureshi AI Caplan LR. Intracranial atherosclerosis. Lancet. (2014) 383:984–98. 10.1016/S0140-6736(13)61088-0 24007975

[18] Kang DH Kim YW Hwang YH Park SP Kim YS Baik SK. Instant reocclusion following mechanical thrombectomy of in situ thromboocclusion and the role of low-dose intra-arterial tirofiban. Cerebrovasc Dis. (2014) 37:350–5. 10.1159/000362435 24941966

[19] Kim BM. Causes and solutions of endovascular treatment failure. J Stroke. (2017) 19:131–42. 10.5853/jos.2017.00283 28592777 PMC5466284

[20] Yoon W Kim SK Park MS Kim BC Kang HK. Endovascular treatment and the outcomes of atherosclerotic intracranial stenosis in patients with hyperacute stroke. Neurosurgery. (2015) 76:680–6; discussion: 686. 10.1227/NEU.0000000000000694 25988927

[21] Lee JS Hong JM Kim JS. Diagnostic and therapeutic strategies for acute intracranial atherosclerosis-related occlusions. J Stroke. (2017) 19:143–51. 10.5853/jos.2017.00626 28592778 PMC5466291

[22] Al Kasab S Almadidy Z Spiotta AM Turk AS Chaudry MI Hungerford JP. Endovascular treatment for AIS with underlying ICAD. J Neurointerv Surg. (2017) 9:948–51. 10.1136/neurintsurg-2016-012529 27502403

[23] Hwang YH Kim YW Kang DH Kim YS Liebeskind DS. Impact of target arterial residual stenosis on outcome after endovascular revascularization. Stroke. (2016) 47:1850–7. 10.1161/STROKEAHA.116.013046 27174525 PMC4927379

[24] Hong MK Park SW Kim JJ Lee CW Park SJ. Comparison of six-month results of coronary stenting versus balloon angioplasty alone in patients with acute myocardial infarction. Am J Cardiol. (1997) 79:1524–7. 10.1016/S0002-9149(97)00185-9 9185647

[25] Steffenino G Dellavalle A Ribichini F Uslenghi E. Coronary stenting after unsuccessful emergency angioplasty in acute myocardial infarction: results in a series of consecutive patients. Am Heart J. (1996) 132:1115–8. 10.1016/S0002-8703(96)90453-6 8969561

[26] Turi ZG McGinnity JG Fischman D Kreiner MJ Glazier JJ Rehmann D. Retrospective comparative study of primary intracoronary stenting versus balloon angioplasty for acute myocardial infarction. Cathet Cardiovasc Diagn. (1997) 40:235–9.3.0. 10.1002/(SICI)1097-0304(199703)40:3<235::AID-CCD1>3.0.CO;2-B 9062712

[27] Levy EI Siddiqui AH Crumlish A Snyder KV Hauck EF Fiorella DJ. First Food and Drug Administration-approved prospective trial of primary intracranial stenting for acute stroke: SARIS (stent-assisted recanalization in acute ischemic stroke). Stroke. (2009) 40:3552–6. 10.1161/STROKEAHA.109.561274 19696415

[28] Goyal M Orlov K Jensen ME Taylor A Majoie C Jayaraman M. A DELPHI consensus statement on antiplatelet management for intracranial stenting due to underlying atherosclerosis in the setting of mechanical thrombectomy. Neuroradiology. (2021) 63:627–32. 10.1007/s00234-020-02556-z 32974691

[29] Yang M Huo X Miao Z Wang Y. Platelet glycoprotein IIb/IIIa receptor inhibitor tirofiban in acute ischemic stroke. Drugs. (2019) 79:515–29. 10.1007/s40265-019-01078-0 30838514

[30] Yang J Wu Y Gao X Bivard A Levi CR Parsons MW. Intraarterial versus intravenous tirofiban as an adjunct to endovascular thrombectomy for acute ischemic stroke. Stroke. (2020) 51:2925–33. 10.1161/STROKEAHA.120.029994 32933416

[31] Baek BH Yoon W Lee YY Kim SK Kim JT Park MS. Intravenous tirofiban infusion after angioplasty and stenting in intracranial atherosclerotic stenosis-related stroke. Stroke. (2021) 52:1601–8. 10.1161/STROKEAHA.120.033551 33793319

[32] Lin YH Chen KW Tang SC Lee CW. Endovascular treatment outcome and CT angiography findings in acute basilar artery occlusion with and without underlying intracranial atherosclerotic stenosis. J Vasc Interv Radiol. (2020) 31:747–53. 10.1016/j.jvir.2019.09.002 32107127

